# Antimicrobial Cyclic Dipeptides from Japanese Quail (*Coturnix japonica*) Eggs Supplemented with Probiotic *Lactobacillus plantarum*

**DOI:** 10.4014/jmb.2311.11006

**Published:** 2023-12-19

**Authors:** Sa-Ouk Kang, Min-Kyu Kwak

**Affiliations:** 1 Laboratory of Biophysics, School of Biological Sciences, and Institute of Microbiology, Seoul National University, Seoul 08826, Republic of Korea; 2 Laboratory of Microbial Physiology and Biotechnology, Department of Food and Nutrition, College of Bio-Convergence, and Institute of Food and Nutrition Science, Eulji University, Seongnam 13135, Republic of Korea

**Keywords:** Cyclic dipeptides, *cis*-cyclo(L-Leu-L-Pro), *cis*-cyclo(L-Ser-L-Pro), multidrug-resistant bacteria, pathogenic fungi, quail eggs

## Abstract

Fifteen cyclic dipeptides (CDPs) containing proline, one cyclo(Phe-Ala) without proline, and a non-peptidyl DL-3-phenyllactic acid were previously identified in the culture filtrates of *Lactobacillus plantarum* LBP-K10, an isolate from kimchi. In this study, we used Japanese quail (*Coturnix japonica*) eggs to examine the effects of probiotic supplementation on the antimicrobial CDPs extracted from quail eggs (QE). Eggshell-free QE were obtained from two distinct groups of quails. The first group (K10N) comprised eggs from unsupplemented quails. The second group (K10S) comprised eggs from quails supplemented with *Lb. plantarum* LBP-K10. The QE samples were extracted using methylene chloride through a liquid-liquid extraction process. The resulting extract was fractionated into 16 parts using semi-preparative high-performance liquid chromatography. Two fractions, Q6 and Q9, were isolated from K10S and identified as *cis*-cyclo(L-Ser-L-Pro) and *cis*-cyclo(L-Leu-L-Pro). The Q9 fraction, containing *cis*-cyclo(L-Leu-L-Pro), has shown significant inhibitory properties against the proliferation of highly pathogenic multidrug-resistant bacteria, as well as human-specific and phytopathogenic fungi. Some of the ten combinations between the remaining fourteen unidentified fractions and two fractions, Q6 and Q9, containing *cis*-cyclo(L-Ser-L-Pro) and *cis*-cyclo(L-Leu-L-Pro) respectively, demonstrated a significant increase in activity against multidrug-resistant bacteria only when combined with Q9. The activity was 7.17 times higher compared to a single *cis*-cyclo(L-Leu-L-Pro). This study presents new findings on the efficacy of proline-containing CDPs in avian eggs. These CDPs provide antimicrobial properties when specific probiotics are supplemented.

## Introduction

The culture filtrates (CFs) of lactic acid bacteria (LAB) contain biologically active and antimicrobial substances [1, 2]. Bioactive CFs primarily consist of low-molecular weight compounds such as organic acids, hydrogen peroxide, carbon dioxide, diacetyl, acetaldehyde, and proteinaceous/non-proteinaceous bacteriocins [3, 4]. 2,5-diketopiperazines (DKPs), the simplest form of cyclic dipeptides (CDPs), and their derivatives have garnered considerable interest in scientific research as a promising source of bioactive compounds for medical purposes. The unique chirality, structural diversity, and potential pharmaceutical applications of cyclotides and related peptide scaffolds make them valuable for drug design [5, 6]. CDPs are highly significant due to their efficacy as antimicrobial agents and their distinctive physiological roles associated with human diseases, such as dermatitis, dementia, diabetes, pancreatic disorders, and neurodegenerative disorders [7, 8]. Cyclo(His-Leu) from *Bacillus subtilis* B38 displays antioxidative properties [9]. Cyclo(L-Pro-D-Arg) from *B. cereus* present in a rhabditid entomopathogenic nematode exhibits antibacterial and antitumor properties and acts as a chitinase enzyme activity suppressor [10]. *Penicillium* sp. F70614 and *P. italicum* FUN2 both produce α-glucosidase inhibitors, specifically cyclo(dehydroAla-Leu) and *cis*-cyclo(prolyl-valyl), respectively [11, 12]. High doses of zinc and cyclo(His-Pro), known for their anti-hyperglycemic properties, effectively lower blood glucose levels by enhancing muscle glucose uptake in both healthy human subjects [13] and obese diabetic (ob/ob) mice [14]. Other histidine-containing CDPs, such as cyclo(His-Phe) and cyclo(His-Tyr), inhibit the proliferation of bacteria and fungi. These CDPs exert their influence on intracellular ion channels, leading to the induction of cell death in various carcinoma cell lines, such as HeLa (cervical), WHCO3 (esophageal), and MCF-7 (breast) [15]. We have previously shown the antimicrobial properties of proline-based CDPs, either alone or in combination, in the filtrates from *Lactobacillus plantarum* LBP-K10 (LBP-K10), *Leuconostoc mesenteroides* LBP-K06 (LBP-K06), and kimchi [16, 17]. Significant progress has been made in investigating the regulatory mechanism that affects CDP biosynthesis, thanks to the interdependent relationships among metabolites synthesized by probiotics [18]. It is widely recognized that *Lactobacillus* spp. have proven to be effective against microbial pathogens, as demonstrated by the antimicrobial activities of LAB [19, 20]. Antimicrobial compounds, specifically CDPs, are present in the CFs of *Lactobacillus* species. These CDPs include different types of diketopiperazines, including the 2,3-, 2,5-, and 2,6-diketopiperazines, and their derivatives. These CDPs have been extensively studied for their ability to inhibit both Gram-positive and Gram-negative bacteria, as well as fungi [21, 22].

The diverse physiological functions of secondary metabolites found in avian eggs encompass a wide range of bioactivities [23-25]. These reports highlight the significance of egg components in preventing and controlling diseases. The biochemical, nutritional, and pharmaceutical characteristics of avian eggs demonstrate the presence of stress-responsive regulatory components and defense mechanisms that are essential for fending off pathogenic microorganisms [26]. Although chicken eggs remain the most commonly consumed, there has been an increasing interest in eggs obtained from a variety of avian species due to their acknowledged nutritional value. This heightened attention towards alternative egg sources can be attributed to their recognized benefits. These eggs possess the potential to enhance physiological functions and display unique bioactive properties [27, 28]. The growing interest in non-traditional avian species is evident in the recent focus on Japanese quail (*Coturnix japonica*) eggs [29]. The composition profiles/ratios of quail eggs (QE) yolk components are significant. QE yolks have higher levels of monounsaturated fatty acids and phosphatidylcholine, as well as lower levels of phosphatidylethanolamine, and a lower ratio of polyunsaturated to saturated fatty acids compared to chicken egg yolks [30]. The results suggest that QE may offer greater health benefits.

This study explores the potential of using antimicrobial CDPs derived from non-fermented animal-based raw materials. It is unclear if any form of CDP or its derivatives can be detected in QE or other avian eggs. This study aims to analyze the impact of probiotic LBP-K10 supplementation on the quality of CDP production in quails, compared to those without supplementation. To study the production of CDP in QE, we monitored the effects of administering probiotics LBP-K10 to improve QE quality and targeted CDPs. Following the supplementation of LBP-K10 to the quail's feed and water, two CDPs, *cis*-cyclo(L-Ser-L-Pro) and *cis*-cyclo(L-Leu-L-Pro), were produced in QE. The impact of probiotic supplementation was shown by comparing CDP production and antimicrobial activity. These findings provide a roadmap for future research and related industries, demonstrating how non-fermented feeding modifications can be implemented.

## Materials and Methods

### Reagents

Dichloromethane (methylene chloride, CH_2_Cl_2_) was used as the solvent for extraction in this study (Merck, Germany). Other chemicals and reagents used for analyzing the produced compounds in QE or testing antimicrobial activity were purchased from Sigma-Aldrich (USA). All solvents used in this study, including water, methanol, and acetonitrile, were of High-Performance Liquid Chromatography (HPLC) grade and were obtained from Thermo Fisher Scientific (USA), unless otherwise specified. Triple deionized water (TDW) from a Millipore water purification system (Millipore, USA) was used to dissolve both the lyophilized CDP fractions and the combined unidentified fractions of the QE.

### CDP Preparation

For the purpose of conducting a comparative analysis using a combined method strategy involving HPLC and gas chromatography-mass spectrometry (GC-MS), the CDPs obtained were extracted from LBP-K10 or LBP-K06, as suggested in previous studies [16, 17]. The extraction process involved isolating the following CDPs: *cis*-cyclo(L-Val-L-Pro), *cis*-cyclo(L-Leu-L-Hyp), *cis*-cyclo(L-Ser-L-Pro), *cis*-cyclo(L-Leu-L-Pro), *cis*-cyclo(L-Phe-L-Pro), and a non-peptidyl DL-3-phenyllactic acid. To compare the CDPs and their enantiomeric analogs in QE obtained from the probiotic LBP-K10 supplementation, we purified CDPs from LBP-K10 CFs. The purified CDPs included cyclo(Tyr-Pro), cyclo(Ser-Pro), cyclo(Leu-Pro), cyclo(Val-Pro), cyclo(Phe-Ala), cyclo(Met-Pro), and cyclo(Phe-Pro).

### Probiotic Strain

One strain of probiotic LAB listed in Table 1 is used. LBP-K10 strain was derived from traditional Korean kimchi, and its characterization was conducted using 16S rDNA sequencing, as previously suggested [31]. For the comparative analysis of QE CDP fractions, LBP-K10 and LBP-K06 were cultured on modified de Man, Rogosa, and Sharpe (mMRS) medium without beef extract [32]. If necessary, the bacterial cells were diluted and cultured on mMRS agar media to determine the number of colony forming units (CFU/ml) [33]. The CDP fractions derived from HPLC peaks of LBP-K10 and LBP-K06 were used to determine the CDP content in QEs or for spiking tests, following the methods described in previous studies [16, 17].

### Pathogenic Strains

All pathogenic bacteria and fungi are listed in Table 1.

We evaluated the antibacterial efficacy of QE fractions and commercial CDPs against Gram-positive and Gram-negative bacteria, including multidrug-resistant strains. The bacterial strains used in this experiment were obtained from the Korea National Institute of Health (KNIH).

To evaluate the antifungal activity, we used human pathogenic *Candida albicans* [34] and phytopathogenic *Ganoderma boninense* isolates (GMR3) [35]. *C. albicans* was grown in minimally synthetic defined (SD) medium, while *G. boninense* was grown in *Ganoderma*-selective medium (GSM) and primarily tested on potato dextrose agar (PDA), as previously recommended [36].

### Ethical Approval

No ethical approval was required for the collection of samples from the tested animals using standard, non-invasive methods that did not cause any harm or distress.

### QE Preparation

The organic quail feed was purchased from Seoul Feed (Republic of Korea). The LBP-K10 probiotic was supplemented at a quail farm in Gyeonggi province, Republic of Korea. A total of 150 quails, aged 14 weeks, were divided into two groups (Table 2): 1) the control group without supplementation, and 2) the experimental quail group with feed supplemented with LBP-K10. The organic feed was formulated to meet or exceed the nutrient requirements for quails as specified by the NRC [37]. This was accomplished without the use of chemicals, prohibited materials, or antibiotics.

To enhance the microbiota with probiotics, we cultured LBP-K10 in mMRS liquid media at 30°C for 72 h. Then, 0.1% of 1 × LBP-K10 cultures were added to the organic feed and drinking water as follows: 100 ml of 1 × probiotic LBP-K10 were dissolved in 900 ml of chlorine-free and disinfectant-free water and left to stand for 24 h. Subsequently, 1 L of probiotic solution was sprayed onto 100 kg of organic feed, resulting in a final probiotic feed concentration of 0.1%. After drying, the probiotics were absorbed, and the feed was prepared for use. To prepare drinking water, 100 ml of a 1 × probiotic LBP-K10 solution were added to 99.9 L of water and mixed thoroughly to generate probiotic water with a final concentration of 0.1%, which is safe to drink. The quails were fed twice daily at 7 a.m. and 5 p.m. for 6 weeks.

### QE Extraction Using CH_2_Cl_2_

75 unfertilized eggs were collected and weighed from quails designated as K10N and K10S in the non-supplemented and probiotic LBP-K10-supplemented feeding groups, respectively (Table 2). The eggshells were cracked, and the contents were frozen and stored at -70°C prior to CH_2_Cl_2_ extraction. To remove proteins, QE samples were treated with 10% trichloroacetic acid (Sigma, USA). The supernatants were filtered using a 0.22 μm-cellulose acetate membrane with an equal amount of TDW. The resulting CFs were extracted using a 10-fold volume of CH_2_Cl_2_ and then evaporated, lyophilized, and dissolved with TDW for HPLC fractionation.

### HPLC Fractionation

We conducted HPLC fractionation on the materials extracted using CH_2_Cl_2_ [16]. The individual compounds and combined fractions were separated into distinct fractions. For QE samples, the extraction was performed using CH_2_Cl_2_ as previously described. This was followed by HPLC fractionation and subsequent re-extraction of the fractions using CH_2_Cl_2_. To avoid non-specific binding of impurities to the Hypersil octadecyl silica (ODS) C18 resin, we filtered and re-separated the QE samples.

The sample fractionation process used a semi-preparative HPLC system (Agilent, USA) with a semi-preparative ODS C18 reverse-phase column (9.4 × 250 mm, Agilent), and ChemStation HPLC software. The initial stage employed a mobile phase consisting of 67% water, 3% acetonitrile, and 30% methanol. This phase ran for 45 min at wavelengths of 210, 260, and 280 nm. The fractions were collected, concentrated, and lyophilized to form a powder.

### Mass Analysis

We used an Agilent GC-MS chromatographic system (USA) to perform electron ionization (EI) and chemical ionization (CI) of each fraction. The system included an Agilent 6890 series GC with a 7679-series automatic liquid sampler and mass analyzer attached to it. Additionally, we used a high-resolution mass spectrometer (Jeol JMS-700, Japan).

### Preparation of CDP-Containing Fractions (QCDPFs)

QCDPFs were prepared by combining various fractions, with some comprising CDPs and others potentially containing unidentified antimicrobial fractions. Antimicrobial assays were performed using five combinations of fractions (*i.e.*, a, b, c, d, and e) in the following procedure: a) individual single CDPs and b) a mixture comprising the *cis*-cyclo(L-Ser-L-Pro)-containing fraction (Q6) with unidentified fractions Q1, Q2, Q^ns1-ns4^ (Q^ns1^, Q^ns2^, Q^ns3^, and Q^ns4^), Q3, Q4, and Q5. c) The fraction containing the *cis*-cyclo(L-Leu-L-Pro)-containing fraction (Q9) was used in the same manner as described in "b," with the exception that fractions Q1, Q2, Q^ns1-ns4^, Q3, Q4, Q5, and *cis*-cyclo(L-Ser-L-Pro) (Q6) were substituted for Q7, Q8, Q10, Q11, Q12, Q13, Q14, and Q15. d) The fraction containing both *cis*-cyclo(L-Ser-L-Pro) (Q6) and *cis*-cyclo(L-Leu-L-Pro) (Q9) was used alongside all other unidentified fractions. e) The fraction containing solely unidentified fractions was also used, but without the two CDP fractions.

All fractions, except for the sample in bullet "a", were categorized as follows. QCDPF1 [Q1, Q2, Q^ns1-ns4^, Q3, Q4, Q5, and Q6 (*cis*-cyclo(L-Ser-L-Pro))], QCDPF2 [Q7, Q8, Q10, Q11, Q12, Q13, Q14, Q15, and Q9 (*cis*-cyclo(L-Leu-L-Pro))], QCDPF3 [QCDPF1 + QCDPF2, including fractions Q6 (*cis*-cyclo(L-Ser-L-Pro)) and Q9 (*cis*-cyclo(L-Leu-L-Pro))], and QCDPF4 [QCDPF1 + QCDPF2, excluding fractions Q6 (*cis*-cyclo(L-Ser-L-Pro)) and Q9 (*cis*-cyclo(L-Leu-L-Pro))], respectively. The CH_2_Cl_2_ extraction was performed on each fraction before and after combining the fractions. The control experiments also involved examining different combined QCDPFs. These included QCDPF5 (QCDPF1 without Q6), QCDPF6 (QCDPF2 without Q9), QCDPF7 (QCDPF3 without Q6), QCDPF8 (QCDPF3 without Q9), QCDPF9 (QCDPF1 with Q9), and QCDPF10 (QCDPF2 with Q6).

To improve sample purity, we performed a set of experiments using sequential HPLC fractionation and CH_2_Cl_2_ extraction at each stage of the procedure. Initially, CH_2_Cl_2_ was used to extract the K10N and K10S samples. Subsequently, individual fractions were obtained through HPLC separation. These fractions were then subjected to a secondary CH_2_Cl_2_ extraction and lyophilization. This process involved secondary modified-HPLC fractionation and re-collection, with at least three repetitions of tertiary re-extraction using five-fold volumes of CH_2_Cl_2_ for each fraction. The fractions were combined by using one fraction for each experiment, selected from the previously designed fractions outlined in bullets a) through e).

### Antibacterial Activity Assays

The fractions isolated from the QE were tested according to previous studies [16, 38]. The antibacterial activity of the samples was evaluated using the National Committee for Clinical Laboratory Standards (NCCLS) [39]. The minimum inhibitory concentration (MIC) was determined by diluting the samples with TDW to concentrations ranging from 400 to 3.125 μg/ml. The incubation was conducted at 37°C for 17 h. The absorbance of the reaction mixture was measured at a wavelength of 600 nm using a microplate reader from Molecular Devices in 96-well plates. The antibacterial efficacy of the isolated fraction was evaluated using bacterial indicators and multidrug-resistant bacteria at a concentration of 5 × 10^5^ CFU/ml.

### Antifungal Activity Assay

The antifungal activity against the pathogenic fungi *C. albicans* and *G. boninense* was determined using the method described [36]. To assess the anti-*Ganoderma* properties of isolated fractions and commercial CDPs, we used six-well PDA plates (3.0 ml) with mycelium and 8.0-mm punctures. We applied a lyophilized fraction (1.5–25 mg) suspended in sterilized distilled water onto the PDA plates using a six-well format. The plates were incubated at 28°C for seven days. The antifungal activity against *C. albicans* was evaluated by inoculating 1 × 10^4^ cells of the wild-type SC5314 into six-well plates with 3 mL of the minimally defined SD agar medium. The plates were incubated at 28°C for 3 days.

### Statistical Analysis

The data are presented as the mean ± standard deviation (SD) of at least three independent experiments to compare the content of CDPs and antimicrobial activities in two samples. The statistical significance of the differences was assessed using the Student's *t*-test in Microsoft Office Excel (2018). Statistically significant differences were assessed using a significance level of *p* < 0.05 (*) for all comparisons.

## Results

### Different Physicochemical Characteristics of QE Samples by Probiotic Supplementation

The study examined the effects of LBP-K10 probiotic supplementation on QE productivity. Neither the K10S with probiotics nor the K10N without supplementation had a significant impact on QE productivity. The lack of effect can be attributed to the fact that both strains were fed the same diet, which had a protein content of 20%(Table 2). K10S showed a slight decrease in the total weight of eggs, representing about 93.94% of the weight observed in K10N. Each set of 75 QE had an average weight of 11.26 ± 0.26 g/1 QE for K10N and 10.58 ± 0.21 g/1 QE for K10S. The study findings indicate that incorporating LBP-K10 supplements as a probiotic had a negligible effect on the weight of QE. However, it did not have a significant effect on their productivity (*p* > 0.05). Additionally, a slight variation was observed in the thickness of the eggshell and the Haugh unit, demonstrating that the consumption of probiotic LBP-K10 led to a slight decrease in weight due to changes in these factors. Table 2 illustrated the optimal experimental conditions for quail feed. The data from both K10N and K10S indicated that probiotic supplementation has minimal effect on feed consumption (g/quail/day), productivity (%, day), average weight (g/egg), eggshell thickness (mm/75), and Haugh unit. The findings suggests that a commercial feed containing 20% protein can meet the NRC requirements as an energy source [37]. Our findings suggest that combining QE with a probiotic supplement may have unexplored physicochemical properties.

### Non-Detectable or Non-Separable Fractions of K10N

**Fig. 1:**
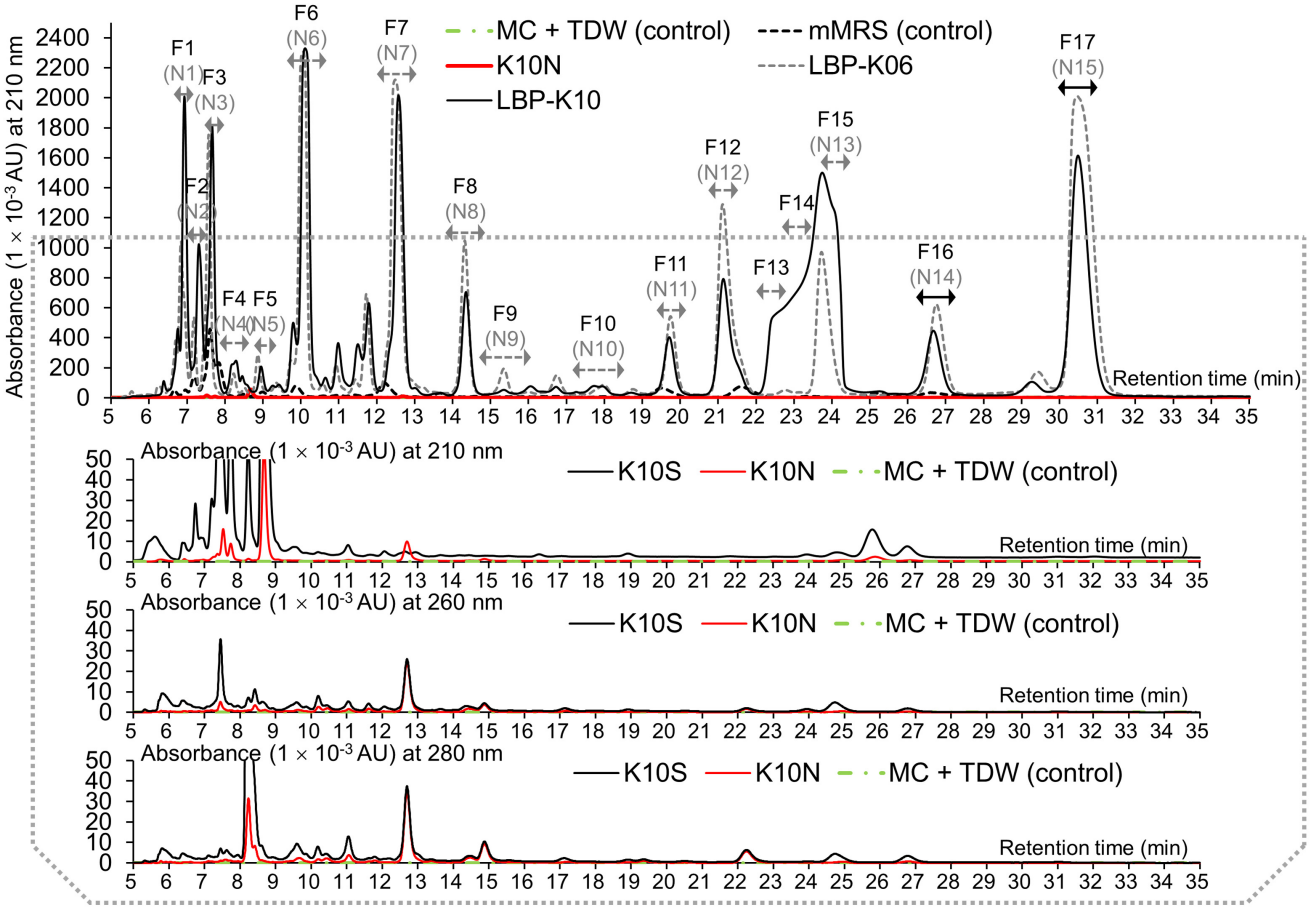
HPLC chromatographic fractionation patterns between K10N and LABs. Fourteen unidentified fractions from K10N are shown. The text specifies that all samples were extracted with CH_2_Cl_2_ in order to achieve HPLC separation. Each experiment was conducted at least five times, with each distinct peak represented by separate lines. The filtrate from the 3-day LBP K-06 or LBP-K10 culture was extracted with CH_2_Cl_2_.

**Fig. 2:**
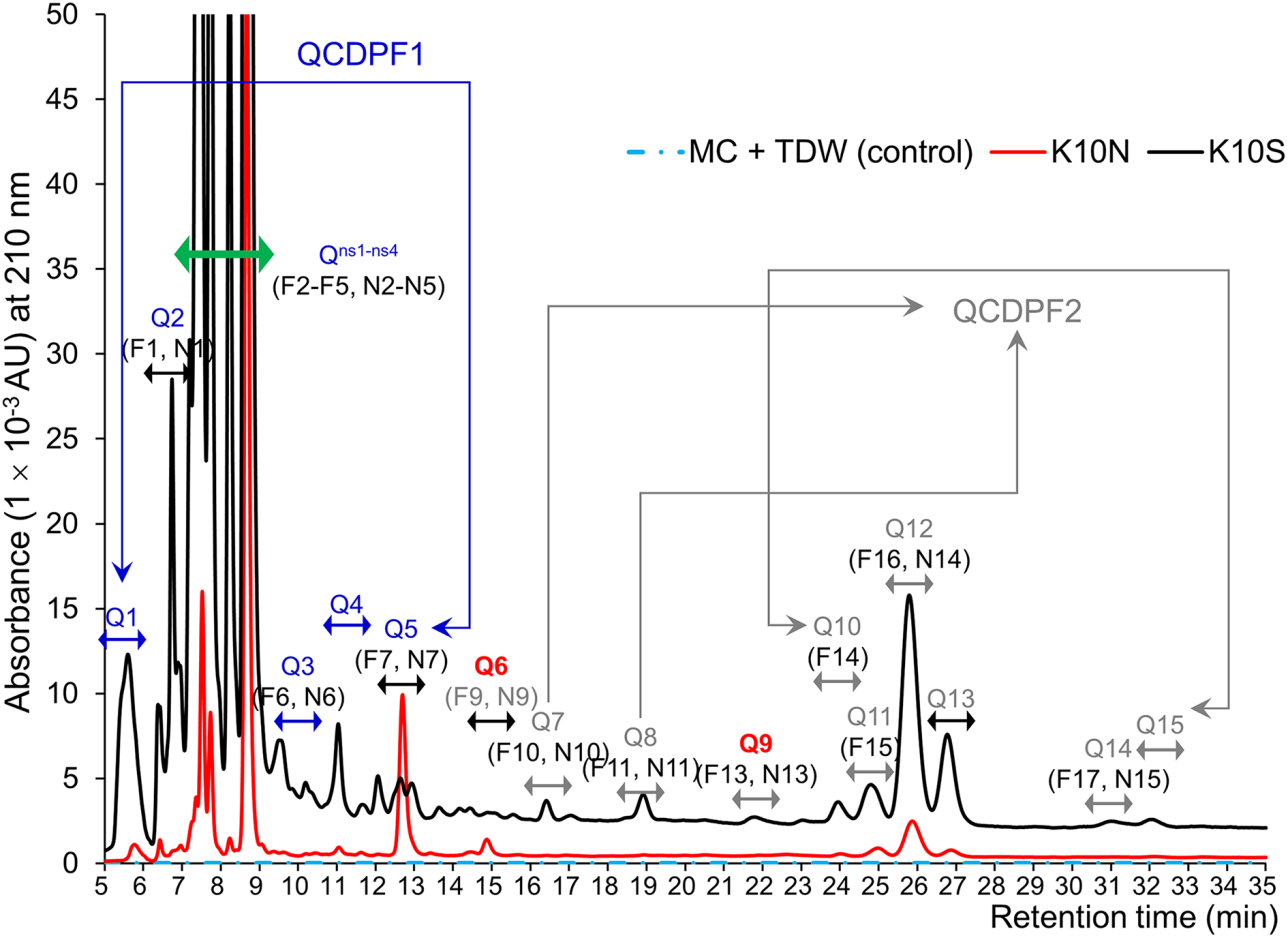
The distinctive chromatographic CDP-like fractions of K10S. The K10N and K10S fractions were observed using HPLC chromatographic fractionation. The experiments were conducted at least three times. The filtrate obtained from a 3-day culture of LBP K-06 or LBP-K10 was used as a reference to compare fractions from plants and animals.

Our experimental approach to identify CDPs in QE involved using analytical HPLC to analyze egg components, as previously described [40]. Additionally, semi-preparative HPLC methods were used to isolate CDPs, as described in our previous study [16]. For both K10N and K10S, we obtained 16 fractions through CH_2_Cl_2_ extraction and HPLC fractionation. The resulting fractions contained inseparable fractions Q^ns1^, Q^ns2^, Q^ns3^, and Q^ns4^ (Fig. 1). The 15 distinct peaks, excluding Q^ns1-ns4^, were observed in both K10N and K10S samples and were designated as Q1 to Q15. The corresponding retention times (*t_R_*) for each peak were illustrated (Figs. 1 and 2, and Table 3). The inseparable peaks, Q^ns1^, Q^ns2^, Q^ns3^, and Q^ns4^, were merged into a solitary fraction denominated Q^ns^. To identify potential CDPs in each QE sample, we separated the K10N sample and compared it to standard CDP fractions F1-F17 in LBP-K10 or N1-N15 in LBP-K06. The comparison of K10N, LBP-K10, and LBP-K06 fractions showed that the use of LBP-K06 peaks was more effective and accurate in isolating a single peak. This phenomenon may be attributed to the presence of broad shoulder peaks in the retention behaviors of LBP-K10, as observed in F13, F14, and F15. The symmetrical and sharp peaks observed in the N1 to N15 range of LBP-K06 were used as reference points for the CDP fractions of K10N or K10S. It was hypothesized that the biophysical characteristics of the candidate CDP fractions in the QE peaks might be similar to those of LBP-K10 fractions.

The HPLC peaks in the K10N chromatograms were identified based on their unique elution order and retention times, which differed from those of LBP-K06. The fractions obtained from K10N showed unclear retention patterns, with some observed peaks among them. The chromatographic resolution patterns of K10N did not align with the observed retention time difference (Δ*t_R_*) in K10S and LBP-K06. Additionally, spiking tests and GC-MS analysis using EI/CI on K10N did not detect any peaks associated with CDPs. The results imply that there is no significant correlation between the proportion of retention peak area in K10N and CDP peaks in LAB. The multiple peaks of K10N were combined mainly due to the significant overlap observed between them. Therefore, further research was needed to determine the factors that impact these variations. Different mobile phase compositions were used to isolate each peak individually in order to accurately estimate the desired parameters. The fractions that were difficult to separate underwent re-chromatography. The study involved using different ratios of methanol (5%, 7.5%, 10%, and 20%), acetonitrile (5%, 10%, and 15%), and water (65-90%). Our attempts to fractionate peaks using isocratic modifications resulted in an increase in the retention volume for each peak. the formation of peaks that increasingly became more difficult to distinguish from one another (Table S1). The CH_2_Cl_2_ extraction-HPLC fractionation did not result in the isolation of any pure compounds through individual fraction separations. Despite our best efforts to separate individual peaks in the HPLC chromatogram using K10N, we were unable to isolate any compounds based on our experimental parameters of elution volume at peak maximum and peak height. This observation underscores the lack of CDP fraction in K10N and accentuates the difficulty in establishing peak-area normalization for precise quantification of impurity fractionation.

### Two Types of CDPs and Their Concentrations in K10S from Probiotic-Supplemented Feed

**Fig. 3:**
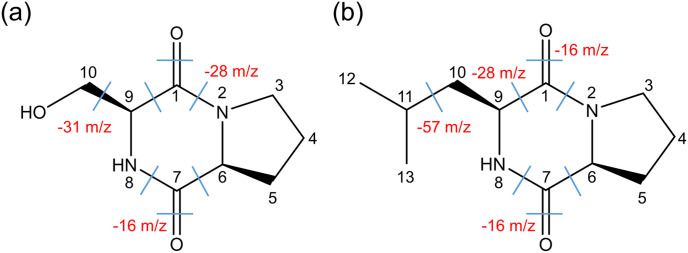
The identified CDPs from fractions Q6 and Q9 of K10S are displayed. The fragmentation patterns based on EI data are shown in Table 4. The separation of structural units by chemical bonds was depicted using dashed lines.

We conducted a modeling study to analyze the dynamic production of CDPs in QE using HPLC peaks in K10S. The retention times of these peaks ranged from 6-8, 7.5-8.5, 9.5-10.5, 22-23.5, and 30.5-31.5 min (Table 3). We employed various isocratic mobile phase solvent combinations to examine K10S, similar to the analysis conducted on K10N. The HPLC retention behaviors of K10S were found to be similar to those of K10N, but with greater distinguishability (Fig. 2). K10S exhibited a noticeable difference in retention time (Δ*t_R_*) for fractions, which aligns somewhat with the reference profiles for LAB CDPs. HPLC peaks with retention time-peak area data matrices, ranging from 14.5 to 15.5 and 22.0 to 23.0 minutes, correspond to Q6 (F9 and N9) and Q9 (F13 and N13), respectively. According to the GC-MS analysis (Table 4), fractions Q6 and Q9 exhibited molecular ions [M+1]^+^ of 185.0 and 211.0, respectively. The results suggest the presence of *cis*-cyclo(L-Ser-L-Pro) and *cis*-cyclo(L-Leu-L-Pro), which can be identified by CI [M+1]^+^ of 185.0 and 211.0, respectively [16]. The EI mass fragmentation data for Q6 and Q9 revealed the existence of two isomers: *cis*-cyclo(L-Ser-L-Pro) and *cis*-cyclo(L-Leu-L-Pro), identified as C_8_H_12_N_2_O_3_ and C_11_H_18_N_2_O_2_, respectively (Table 4 and Fig. 3). Additionally, fragmentation patterns Q6 and Q9 were observed in the EI-MS spectra of proline-based DKPs, typically with an m/z value of 154 or 155. The quantities of Q6 and Q9, which are *cis*-cyclo(L-Ser-L-Pro) and *cis*-cyclo(L-Leu-L-Pro) respectively, were found to be 5.33 (0.29 ± 0.01 mg/l) and 7.41 (0.44 ± 0.02 mg/l) times lower than F9 and F13 of LBP-K10. The amounts of Q6 and Q9 were lower than N9 and N13, respectively. This indicates a limited CDP pool in QE even with probiotic supplementation.

### The Antibacterial Efficacy of the Isolated Fractions and no Antibacterial Activity in the Unidentified Fractions

The in vitro assessment of the antibacterial and probiotic potential of each fraction showed minimal activity against various bacterial indicators (Table 5). The unidentified K10S fractions from Q1 to Q15, including Q^ns^, exhibited minimal to no antibacterial activity against bacterial indicators and multidrug-resistant bacteria in the MIC test. The data from the extensively performed MIC tests, except for Q9, indicate that there is no correlation between the unidentified animal-derived fractions in QE and antimicrobial activities. The active concentrations for the anti-*Ganoderma* activity of Q6 and Q9 were found to be 5.63 ± 0.91 and 4.98 ± 0.63 (*p* < 0.05), respectively. However, there was no evidence of anti-Candida activity (Table 6 and Table S2). The Q6 and Q9 fractions have targeted antibacterial and antifungal effects against an extensive range of pathogens. Specifically, fraction Q9 containing *cis*-cyclo(L-Leu-L-Pro) exhibited the highest potency against both microbial targets among the fractionated fractions.

The fraction Q9 has been found to possess anti-*S. aureus* properties, with MIC values of 11.55 ± 0.62. Conversely, Q6 did not display any antibacterial activity. These findings are consistent with prior research, especially studies that have used *cis*-cyclo(L-Leu-L-Pro) [16, 17]. The antimicrobial activity data from fraction Q9 shows similarities with other microorganisms, such as *Lactobacillus* isolates, *Streptomyces* sp. KH-614, and *Achromobacter xylosoxidans* [41-43]. Fraction Q9 demonstrated the highest antibacterial activity against multidrug-resistant bacteria (Table 5). Our findings support previous research on antimicrobial CDPs produced by LAB [11, 42, 44, 45]. The MIC values against multidrug-resistant bacteria align with the growth effects of cyclo(L-leucyl-L-prolyl) and cyclo(L-phenylalanyl-L-prolyl) on five strains of vancomycin-resistant enterococci and pathogenic yeasts [46]. Cyclo(Leu-Pro) from LBP-K06 expresses similar effects on multidrug-resistant Gram-positive and Gram-negative bacteria, as previously reported [17]. The MIC test against bacterial indicators showed that the previously established *cis*-cyclo(L-Leu-L-Pro) fractions F13 [16] and N13 [17] from LBP-K10 and LBP-K06, respectively, had MIC values of 10.1 mg/l and 13.55 mg/l against *Bacillus subtilis*, 13.50 mg/l and 12.06 mg/l against *S. aureus*, and 9.7 mg/l and 10.41 mg/l against *Escherichia coli*. The MIC values of fractions F13 and N13 were similar to our current study using fraction Q9. The MIC values against *B. subtilis*, *S. aureus*, and *E. coli* were found to be 11.6 mg/l, 13.0 mg/l, and 10.0 mg/l, respectively. In previous studies on multidrug-resistant bacteria, fractions F13 and N13 exhibited MIC values of 22.40 mg/l and 17.28 mg/l, respectively, against *S. aureus* 11471, and 11.6 and 18.19 against *Salmonella* Typhimurium 12219. The MIC values of fractions F13 and N13 were consistent with our current study using fraction Q9. They exhibited inhibitory effects of 23.0 mg/l and 14.0 mg/l against *S. aureus* 11471 and *S*. Typhimurium 12219, respectively. The similar MIC results observed for fractions LBP-K10 F13, LBP-K06 N13, and QE Q9 can be attributed to the fact that both fractions Q6 and Q9 were found to be pure and single fractions of *cis*-cyclo(L-Ser-L-Pro) and *cis*-cyclo(L-Leu-L-Pro) through our structural analysis.

### Enhanced Antimicrobial Activity of QCDPFs Compared to Single Fraction Q9

The combined QE fractions, QCDPFs, were utilized following the protocol specified in the Materials and Methods section. Among the QCDPFs, QCDPF3 comprised QCDPF1 (7 QE fractions) and QCDPF2 (9 QE fractions) resulting in QCDPF3 containing fractions Q6 (*cis*-cyclo(L-Ser-L-Pro)) and Q9 (*cis*-cyclo(L-Leu-L-Pro)). Additionally, QCDPF7 was formed as a subset of QCDPF3 minus Q6 (*cis*-cyclo(L-Ser-L-Pro)). Both fractions (QCDPF3 and QCDPF7) containing Q9 demonstrated significantly higher antibacterial and antifungal activity compared to the other QCDPFs (Table 6). The Q9 fraction maintained its efficacy at 23.0 mg/l, 21.0 mg/l, and 14.0 mg/l concentrations against multidrug-resistant strains, including *S. aureus* 11471, *S. pneumoniae* 14596, and *S*. Typhimurium 12219. Particularly significant were the antibacterial effects of QCDPF3 when compared to fraction Q9 alone on all tested bacteria. The active concentrations of QCDPF3 (QCDPF1+QCDPF2) demonstrated a 1.58-, 1.67-, and 1.74-fold increase in resistance against the examined multidrug-resistant bacteria relative to the Q9 single fraction. However, the active levels of *cis*-cyclo(L-Leu-L-Pro) in QCDPF3, which is a combination of QCDPF1 and QCDPF2, were measured to be 3.21, 3.11, and 2.01 mg/l against multidrug-resistant strains, including *S. aureus* 11471, *S. pneumoniae* 14596, and *S*. Typhimurium 12219. The MIC values of single fraction Q9 ranged from 23.0 to 14.0 mg/l, which were significantly higher than the active *cis*-cyclo(L-Leu-L-Pro) content of fraction QCDPF3. Based on the MIC data, QCDPF3 demonstrated significantly higher efficacy in reducing the concentration of active *cis*-cyclo(L-Leu-L-Pro) compared to the single use of Q9 fraction. The decrease in concentration was 7.17-fold, 6.75-fold, and 6.97-fold against multidrug-resistant strains, including *S. aureus* 11471, *S. pneumoniae* 14596, and *S*. Typhimurium 12219. Interestingly, the QCDPF7 (QCDPF3 without Q6 (*cis*-cyclo(L-Ser-L-Pro))) exhibited comparable antibacterial activity to QCDPF3 against multidrug-resistant strains, such as *S. aureus* 11471, *S. pneumoniae* 14596, and *S*. Typhimurium 12219.

Treatment with QCDPF3 and QCDPF7 demonstrated a stronger inhibitory effect on the growth of *G. boninense* and *C. albicans* compared to using single fractions Q6 and Q9. This aligns with the antibacterial MIC findings. The data showed that the effective concentrations of QCDPF3, a fungi inhibitor, are significantly lower than the previously reported MIC values of 20 mg/mL for cyclo(L-Ile-L-Pro) [47], as well as the concentrations of cyclo(L-Phe-L-Pro) reported for *Fusarium sporotrichioides* and *Aspergillus fumigatus* [44]. Our study found that QCDPF3 exhibits a stronger antifungal activity compared to other QCDPFs and single CDPs. The specific mechanism responsible for the difference in antifungal activity between *cis*-cyclo(L-Ser-L-Pro) and *cis*-cyclo(L-Ser-L-Pro)-containing fraction QCDPF1, in comparison to fractions Q9, QCDPF3, and QCDPF7, could not be determined. To support our findings, we evaluated the antimicrobial efficacy of additional combined fractions, both with and without fraction Q9 (Table 6). No antimicrobial activity was found in Q6-containing QCDPF1 or any Q9-eliminated QCDPFs, which is in stark contrast to the Q9-containing QCDPFs. When the QCDPF lacked Q9, the sample demonstrated no activity against multidrug-resistant bacteria or pathogenic fungi. The presence or absence of fraction Q6 did not affect the antimicrobial effect of all QCDPFs. The study encountered difficulties due to the low concentrations of Q6 and Q9 fractions, as well as the absence of other diastereoselective/enantiomeric CDPs in K10N and K10S. However, our findings offer compelling evidence that probiotic supplementation leads to the production of CDPs. This is accomplished by combining antimicrobial fractions containing CDP with non-bioactive fractions that have a high selectivity for CDPs.

## Discussion

Our study investigates the antimicrobial effects and potential industrial uses of avian CDPs in combination with probiotics, emphasizing their enhanced capabilities. Non-fermented QE is an experimental material that distinguishes itself apart from microorganisms due to its inability to ferment. The addition of probiotics stimulates the production of highly bioactive CDPs in avian eggs. These probiotic supplements can improve the physiological functions of avian eggs, leading to attributes such as antiadhesive, antioxidant, antimicrobial, anticancer, immunomodulatory, and antihypertensive properties [23-25]. Additionally, the bioactive, nutritional, and medicinal properties of avian eggs suggest the potential to improve stress-responsive regulatory elements and defensive mechanisms against microbial pathogens through probiotic supplementation that produces CDPs. Probiotic supplementation enhances the production of CDP and introduces a methodology for obtaining CDP-rich byproducts with enhanced bioactivity. We observed the presence of two proline-containing CDPs following probiotic supplementation in the K10S experimental group, compared to the K10N control group. The pivotal experiment in this study is Table 6, which examines the impact of probiotic supplementation on specific CDP synthesis and their combinations. To support our findings, we propose ten QCDPFs based on the K10S fractions in the presence and absence of Q9, to achieve higher antimicrobial efficacy. This experimental design provides compelling evidence for the selectivity of bioactive CDPs by using a combination of antimicrobial CDP-containing fractions and non-bioactive fractions.

However, during the coupled CH_2_Cl_2_ extraction and HPLC process, K10N fractions exhibit elusive and inseparable peaks that do not contain CDP fractions. The fractions from K10N show significant disparities from the bioactive CDP fractions found in K10S. This finding contradicts the microbial production of CDP in LAB cultures, starter kimchi, and various Gram-positive and -negative bacteria [11, 22, 42, 43, 47, 48]. The chromatographic analysis is unable to detect and measure the K10N fractions accurately due to vague chromatographic peaks. This leads to low resolution and imprecise quantification, including overlapping or shifted elution times and peaks with a low signal-to-noise ratio. The behavior displayed by K10N can be likened to the chromatographic profiles observed in specific fermented plant materials [17]. Upon further analysis of the chromatograms, it appears that K10N does not contain the basic type of CDPs, indicating the absence of the biosynthetic pathway for 2,5-DKPs. The K10N enzyme, derived from the naturally-fed group, seems to have difficulty forming two cis-amide bonds that are typically produced by the combination of two α-amino acids [21]. Our study indicates that naturally fed quails do not produce CDP-like substances, as they lack the ability to produce bioactive CDPs.

**Fig. 4:**
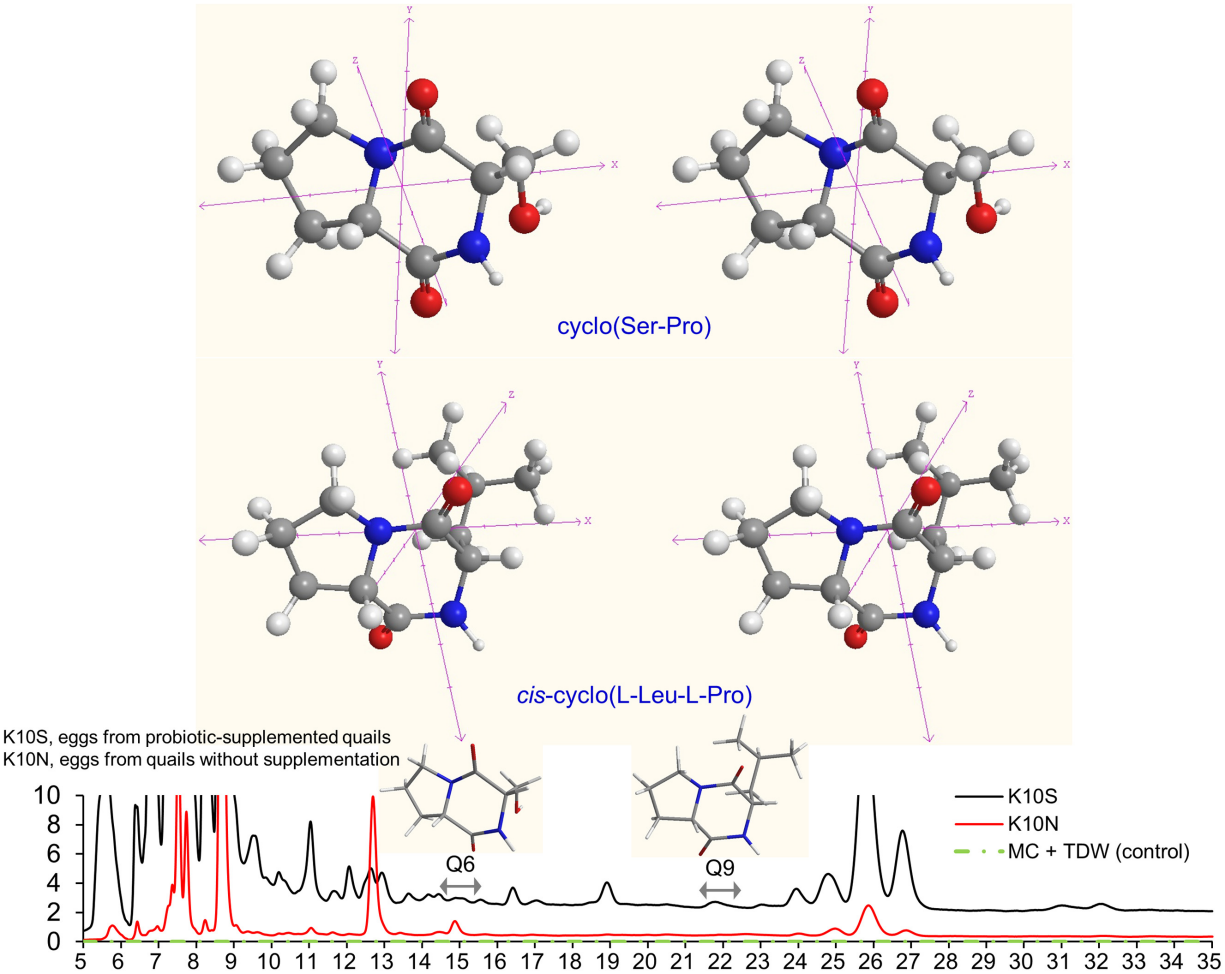
QE CDPs were obtained from active fractions Q6 and Q9 of K10S. The fragmentation patterns of EI are shown in Fig. 3. The separation of structural units by each chemical bond was identified using dashed lines. The threedimensional structures of *cis*-cyclo(L-Ser-L-Pro) (top), *cis*-cyclo(L-Leu-L-Pro) (middle), and their corresponding fractions (bottom) are presented. Each stereo presentation was displayed based on its corresponding label. Carbon atoms were represented as grey, hydrogen as white, oxygen as red, and nitrogen as blue.

The data from Table 3 shows that the K10S contains compounds in various forms. Based on the distinct peak shapes and distribution in K10S, this sample is crucial for detecting the potential presence of CDPs and determining if they are affected by probiotic supplementation. Table 4 and Fig. 4 support the findings of previous studies on EI fragmentation in different food materials and microbes [17, 31, 36, 44, 49-52]. However, it is not feasible to obtain the majority of K10S fractions through HPLC fractionation due to the same issue observed in K10N. Inconsistent molecular ion peaks with the previously established ones result in overlapping and inseparable peaks. The results of K10S fractions do not consistently align with the previously identified molecular ion peaks, although they do show some similar retention characteristics to LAB CFs. Our analysis using GC-MS EI/CI on the K10S fractions was unable to fully characterize them. However, we did find that fractions Q1, Q2, Q8, Q9, and Q11 correspond to bacterial strains F1, F5, F14, F16, and F17 (N15). The supplementation of probiotic LBP-K10 confirms the presence of only two proline-based CDPs (Q6 and Q9) in the K10S.

Figs. 1 and 2, along with Table 3, display 16 fractions (Q1 to Q15, including Q^ns1-ns4^) and their corresponding retention times (*t_R_*). The study employs a fractionation strategy that combines CH_2_Cl_2_ extraction with HPLC fractionation and multiple repetitions to isolate target compounds. This method enables the isolation of pure individual CDPs for analysis using GC-MS, effectively eliminating other peptidyl or non-peptidyl compounds and impurities. Our experimental method convincingly indicates CDP fractions in avian eggs. The CH_2_Cl_2_ extraction of the QE is a highly selective purification method for CDPs, which is consistent with our previous research [16, 17, 31, 36]. The results of screening studies on the QE CDPs indicate a potential association with the probiotic effect previously shown by *Lb. plantarum* WCFS1, based on data derived from the probiotic strain LBP-K10 CDPs. *Lb. plantarum* has the ability to dissipate pyruvate and acts as a fermenter with notable nutritional and bioactive properties [53, 54]. The in vitro analysis shows that *Lactobacillus* spp. exhibit antagonistic activity against a wide range of pathogens and have various physiological activities, highlighting their potential as probiotics with functional advantages. These properties include cholesterol and nitrate removal, free radical scavenging, immune response stimulation, and high exopolysaccharide production [18]. The study's results align with previous screening studies on CDP that also used *Lb. plantarum* sourced from different types of kimchi. These findings demonstrate that these strains exhibit superior levels of antimicrobial activity compared to other isolates [55-57]. The data presented further supports the antimicrobial properties of CFs derived from *Lb. murinus* AU06 against pathogenic bacteria that can cause diseases in fish. Another study showcases the antimicrobial properties of *Lb. plantarum* strain LR/14 against *C. albicans* and *Lb. plantarum* LD1 against Gram-positive and Gram-negative bacteria isolated from marine sediments, the rhizospheric region, and Indian fermented food [58].

Figs. 1, 2, and Table 3 elucidate the effectiveness of CH_2_Cl_2_ extraction for purifying CF in CDPs, as previously discussed [17]. According to previous research, bacteriocins and bacteriocin-like substances found in the cell-free supernatants of various LAB have shown antimicrobial effects [59, 60]. Following multiple HPLC separations and CH_2_Cl_2_ extractions, all isolated CDPs are pure enough for analysis as a singular compound using EI/CI GC-MS. No other substances or contaminants were detected under any experimental condition. UV spectroscopy is a commonly used method to measure the concentration and purity of biomolecules, including peptides, proteins, nucleotides, RNA, and DNA, in order to ensure compound purity [61]. Sample concentration and purity can be determined by measuring the absorbance at specific wavelengths. For peptides, the absorbance at 210 nm is used [62], for proteins it is 280 nm [63], and for nucleotides it is 260 nm. However, the detection limits for longer chain length CDPs are reduced because the UV extinction coefficient at 210 nm increases [64]. The solubility of cyclic peptidyl molecules may decrease as the chain length increases, which can result in errors. It is unlikely that the oligomer produced exceeded the aqueous solubility limit at room temperature. The absorbance at 280 nm is caused by the presence of aromatic amino acids, such as tyrosine, phenylalanine, and tryptophan [63]. Nucleotides contain purines and pyrimidines with conjugated double bonds, which absorb UV absorbance at 260 nm [65]. The OD 260 to OD 280 ratio is commonly used to assess the purity of nucleotide samples by measuring the level of protein contamination. The OD 260/OD 230 ratio can indicate the presence of salts or organic compounds in the sample. The hyperchromic effect can be utilized to test nucleotide pairing using UV spectroscopy. Paired nucleotides exhibit lower UV absorbance compared to unpaired nucleotides, resulting in a decrease in OD 260 when single-stranded DNA or RNA forms double-stranded structures. The light-absorption properties of the pentapeptide hydrogel can be observed by analyzing its UV-Vis spectrum in water. Pentapeptides have distinct absorption traits in the far-UV (<220 nm) and near-UV (280 nm) spectra. The absorption peak observed below 220 nm indicates both π ↔ π* transitions of the peptide bond [66, 67] and the carboxylic acid groups in the peptide [68, 69]. The absorption peak at 280 nm is caused by the aromatic side chains of Trp, while a less intense shoulder at 292 nm is observed due to a weaker π ↔ π* transition.

Our previous study identified the diastereoselective or enantiomeric structures found in the 16 naturally occurring CDPs from LBP-K10 [16]. The analyzed QE CDPs in this study are believed to exhibit bioactivity that depends on conformational fluctuations. The GC-MS EI/CI analysis suggests that specific fractions of LBP-K10 contain the same CDPs, even though their retention behaviors (*t_R_*) may vary. These structural variations have antimicrobial properties and result from intramolecular hydrogen bonding between the carboxy and carboxyamide groups in the cyclic dipeptidyl moiety. This process involves the heterocomplexation of a chiral dipeptide and results in the quantitative enrichment of enantiomers [50, 70]. The potential interaction with biologically active chiral dipeptides often results in the production of large quantities of enantiomers from LAB CDPs. Differences in the Δ*t_R_* of the QE CDPs are anticipated due to the presence of potential conformational enantiomeric pairs or diastereomers. The QE CDPs obtained in this study are hypothesized to demonstrate bioactivities that depend on conformational variations. The QE, which is derived from animal material, has significantly lower amounts of CDP compared to other substances. This is because of its unique retention properties, which do not include conformational enantiomeric pairs or diastereomers. Based on the antibacterial activity data from Table 5, it appears that the bioactive potency of K10S, which includes CDPs and unidentified components, may be relatively low or even absent compared to LAB CFs. The advantage of microbial filtrates over the QE feature is considered to be less significant when compared to the enhanced bioactivities of combined fractions containing microbial CDPs [16]. Our study presents novel research on proline-containing CDPs in avian eggs. The investigation was motivated by discrepancies between our QE data and prior findings for specific strains of *Lactobacilli* and *Leuconostoc*, as well as the previously reported broad-spectrum antimicrobial effectiveness of 17 LBP-K10 fractions. These CDPs have antimicrobial and antiviral properties due to targeted probiotic supplementation. Therefore, we propose their potential as a new approach for promoting animal health and ensuring food safety. The comparisons between LAB CFs and QE reflect considerations of generating or interconverting in diastereoselective or enantiomeric manners, which are influenced by pH changes and/or acylation/alkylation of the chiral enolates. Another consideration is the production of significant amounts of bioactive CDPs, such as LABs.

This study addresses the limited availability of CDPs and the absence of diastereoselective/enantiomeric synthesis by using modified combined QE fractions as an important coping mechanism. The experimental design outlined in Table 6 combines isolated CDP fractions with unidentified fractions. Our trial using combined fractions suggests a potential new method for advanced mass production or enhanced bioactivity technology. Thus, combinations of uncharacterized fractions, with or without QE CDPs, are used to establish the effectiveness of the combined QE fractions. These subsequent experiments do not require trial-and-error to remove organic acids and/or sugars. Additionally, the near-neutral pH of the QE, ranging from approximately 6.8 to 7.3, does not affect antimicrobial assays due to its properties. The pH levels of K10N and K10S are also similar. The pH properties of the QE make it suitable for biochemical and biophysical analysis, improving the accuracy of assessments by preventing loss due to impurities. As a result, QE experiments eliminate the need for the time-consuming process of combining CH_2_Cl_2_ extraction-ion exchange chromatography after the initial fractionation strategy. This process, which typically requires multiple repetitions for LAB CFs to obtain sufficient quantities of complete CDP sets, involves linking CH_2_Cl_2_ extraction with HPLC fractionation [16].

The antimicrobial activity of the CDPs has not been sufficiently proven based on their structural characteristics. Additionally, it is important to consider the factor of cytotoxicity in the debates surrounding bioactive structures. The antiviral activities of *cis*-cyclo(L-Leu-L-Pro) and *cis*-cyclo(L-Phe-L-Pro) were found to have minimal cytotoxicity at different concentrations, as determined using the 3-[4,5-dimethyl-2-thiazolyl]-2,5-diphenyl-2H-tetrazolium bromide (MTT) assay [31]. The viability of host cells is slightly affected by high concentrations of the two CDPs, especially when the concentrations exceed 10.0 mM. The concentrations of CDPs effective against the influenza virus are significantly lower than those that are cytotoxic to host cells. Normal cell growth is not hindered at high concentrations of the CDPs. Another study using the MTT assay demonstrated the cytotoxic effects of cyclo(-Pro-Tyr) on HepG2 cells. The study findings show that cyclo(-Pro-Tyr) causes dose-dependent cytotoxicity in HepG2 cells. No significant toxicity was observed in mouse Fibroblast McCoy cells at the tested concentrations [71]. A study on the cell viability of three CDPs, cyclo(L-Leu-D-Arg), cyclo(2-hydroxy-Pro-L-Leu), and cyclo-(L-Pro-L-Val)) found that cyclo(L-Leu-D-Arg) exhibits the highest cytotoxicity, with cyclo(2-hydroxy-Pro-L-Leu) closely behind [72]. Cyclo(L-Leu-D-Arg) displays the highest activity against MDAM-B231, a breast cancer cell line, with an IC_50_ of 25 μM. Additionally, the compound shows promising activity against the A549 lung cancer cell line, with an IC_50_ of 50 μM. Cyclo(2-hydroxy-Pro-L-Leu) shows activity against MDAM-B231 with an IC_50_ of 100 μM. However, these three diketopiperazines do not show any toxicity towards normal human fibroblast cells (FS) at concentrations up to 50 μM. This study contributes to the understanding of CDPs from probiotic strains as potential sources for new drugs in the pharmaceutical industry. These CDPs have shown promise, as potent antibacterial and antifungal agents.

Current technology is vulnerable to bacterial infections due to the limited use of LABs as antibacterial agents. Both domestic and international instances have successfully isolated, purified, identified, and commercialized LABs as pure substances containing nisin. Despite being a pioneering product, nisin's limited antibacterial activity range hinders its efficacy as a food preservative in fulfilling its intended purpose. Food products often incorporate antibacterial substances from LABs by utilizing all components of the fermentation liquid. No comprehensive domestic or international research and development has been conducted to pinpoint and isolate the genetic expression of antibiotic substances found in specific food materials-based CDPs as potential alternatives to antibiotics. These extracts have specific uses and applications, rendering them potentially valuable for industrial purposes. No efforts have been made to ascertain the structure of the substance, isolate and purify it, or assess its practical efficacy. In order to develop antibiotic alternatives for human and animal use, it is crucial to isolate and identify the expression genome of LABs that produce the active ingredient found in food materials-based CDPs. Efficiently utilizing and identifying mechanisms for high concentration expression is crucial for maximizing output. The product needs to be isolated and purified to obtain a high-purity yield. This is followed by thorough identification of substance and efficacy to ensure successful product development.

The future appears promising for products containing LABs and metabolites, which are widely used in various forms such as food, probiotics, and medications because of their proven effectiveness and safety. Recently, antibacterial purified substances have been authorized in Japan, following Europés lead. Various industries are currently working towards industrializing these substances, highlighting their substantial potential for commercialization. The advancement in genome isolation and analysis technology, material fermentation, and material structure analysis greatly facilitates the strategy and research and development of antibacterial compounds derived from food materials-based CDPs. With the escalating use of antibiotics has led to a growing need for alternative medical treatments. This is mainly because of the emergence of superbugs and frequent outbreaks of infectious diseases. There is an increasing recognition of the benefits of functional foods that boost immunity and the demand for non-toxic natural preservatives. It is anticipated that in the future, there will be new industrial techniques using LABs or food materials-based CDPs as a source of antibacterial substances. The industrial development of research findings includes the development of alternative antibiotics for human and animal medicine, functional foods, and food preservatives.

## Conclusion

Eggs obtained from quails through probiotic supplementation contain two fractions with CDPs, Q6 (*cis*-cyclo(L-Ser-L-Pro)) and Q9 (*cis*-cyclo(L-Leu-L-Pro)), as well as other unidentified fractions without CDPs. This method does not exhibit diastereoselective alkylation of the chiral enolates derived from CDPs found in the QE fractions, which is in stark contrast to fractions of LAB CFs. The peaks observed in the chromatogram obtained from K10N and K10S cannot be classified as pure and separate constituents since it is unfeasible to consider the isolated fractions as a single compound under all experimental conditions. This study on the QE CDPs establishes a laboratory framework for future research on avian eggs and animals. Probiotic supplementation in quails induces CDPs in avian eggs. In order to obtain more reliable and applicable findings on CDPs in QE and other animal sources, it is necessary to have sufficient quantitative data support from previous studies on well-studied CDPs from microbial sources and fermented plant products, as exemplified in the current study. This study does not consider the potential presence of other bacteriocin-like substances, immune-regulating proteins, and enzymes in avian eggs, as determined by 2D LC-MS/MS ESI at high or low resolution. Only a small amount of CDPs are obtained, which are unlikely to be comparable to the high abundance found in bacterial cultures, CFs, and kimchi. The CDPs identified in the QE exhibit similarities to the proline-containing CDPs typically found in LBP-K10 and LBP-K06. QE experiments do not necessitate the additional and time-consuming process of combining CH_2_Cl_2_ extraction-ion exchange chromatography following the initial fractionation approach. This approach connects CH_2_Cl_2_ extraction with HPLC fractionation and often requires multiple repetitions to obtain enough complete CDP sets. QE CDPs provide new evidence that animal-derived CDPs have a distinct chromatographic fractionation pattern compared to the commonly recognized racemic diastereomers that can be extracted by CH_2_Cl_2_ during microbial fermentation. Our data offers convincing evidence that probiotic supplementation in animals can reliably isolate and identify small bioactive compounds. This report discusses the discovery of powerful antimicrobial compounds found in avian eggs, suggesting their potential for use as antimicrobials and in other applications. Further investigation is needed to determine the specificities of certain CDPs in different probiotic combinations. This is anticipated to generate new markets or alternative product markets for existing antibiotics used in both human and animal medicine.

## Abbreviations

CDPs, cyclic dipeptides; CI, chemical ionization; DKPs, diketopiperazines; EI, electron ionization; FA, fatty acid; GC-MS, gas chromatography-mass spectrometry; HPLC, high-performance liquid chromatography; LAB, lactic acid bacteria; LBP-K10, *Lactobacillus plantarum* LBP-K10; LBP-K06, *Leuconostoc mesenteroides* LBP-K06; CH_2_Cl_2_, methylene chloride; MIC, minimum inhibitory concentration; MRS, de Man, Rogosa and Sharpe agar; ODS, octadecyl silica; QE, quail eggs; TDW, triple-distilled water

## Supplemental Materials

Supplementary data for this paper are available on-line only at http://jmb.or.kr.



## Figures and Tables

**Table 1:** Microbial strains used in this study, including LABs, multidrug-resistant bacteria, bacterial indicators, and pathogenic fungi.

Strain	Types or strains	Source or reference
LAB strains		
*Leuconostoc mesenteroides* LBP-K06	Original isolate from fermented Chinese cabbage	[31]
*Lactobacillus plantarum* LBP-K10	Original isolate from fermented Chinese cabbage	[31]
^a^ Multidrug-resistant bacteria		
Gram-positive bacteria		
*Staphylococcus aureus* 11471	Oxacillin-resistant *S. aureus* (ORSA) 11471, which is resistant to beta-lactam antibiotics, including penicillins (methicillin, dicloxacillin, nafcillin, and oxacillin) and cephalosporins	[16, 17], KNIH
*Streptococcus pneumoniae* 14596	*Streptococcus pneumoniae* 14596, which is resistant to penicillin, erythromycin, tetracycline, and clindamycin	[16, 17], KNIH
Gram-negative bacteria		
*Salmonella* Typhimurium 12219	*Salmonella* Typhimurium 12219, which is resistant to ACSSuT (ampicillin, chloramphenicol, streptomycin, sulphonamides, and tetracycline)	[16, 17], KNIH
^b^ Bacterial indicator strains		
Gram-positive bacteria		
*Bacillus subtilis*	*Bacillus subtilis*	[16, 17]
*Staphylococcus aureus*	*Staphylococcus aureus*	[16, 17]
Gram-negative bacteria		
*Escherichia coli*	*Escherichia coli*	[16, 17]
Pathogenic fungi		
*Candida albicans* SC5314	Wild type isolate	[34]
*Ganoderma boninense* GMR3	Wild type isolate	[36]

The antimicrobial and antiviral efficacy of QE fractions was evaluated using these microbes.

The KNIH provided ^a^multidrug-resistant and ^b^indicator bacteria used in this study.

**Table 2:** Overall characteristics of QEs obtained from probiotic-supplemented and non-supplemented feed.

Sample	Treatment type		Source or feed reference
Japanese quail (*Coturnix japonica*)	Probiotic supplementation		Organic feed and drinking water
	No treatment	*Lb. plantarum* LBP-K10	
	^a^ N.A. /75 quails/6 weeks	0.2%/75 quails/6 weeks	20% protein content in both experiments (NRC standard application)
	Sample type		
QE	K10N	K10S	
Feed consumption (g/quail/day)	22.95 ± 0.38	22.03 ± 0.20	6 week-product with and without LBP-K10
Productivity (%, day)	53.67 ± 0.65	52.92 ± 0.81	6 week-product with and without LBP-K10
Average weight (g/egg)	11.26 ± 0.26	10.58 ± 0.21	6 week-product with and without LBP-K10
Egg shell thickness (mm/75)	17.35 ± 0.16	17.87 ± 0.26	6 week-product with and without LBP-K10
Haugh unit	87.05 ± 0.74	87.14 ± 0.63	6 week-product with and without LBP-K10

^a^Not applicable.

**Table 3:** Retention time (*t_R_*) using HPLC chromatographic separation of K10N and K10S, compared to LBP-K10 and LBP-K06.

QE CDP profile based on LAB CDPs								
Sample retention behavior (t_R_)								
QE			LAB CFs				Predicted molecules using LBP-K10 or LBP-K06	Source or reference
	K10N	K10S		LBP-K10		LBP-K06		
Fraction	^a^ R.T.	R.T.	Fraction	R.T.	Fraction	R.T.		
Q1	^b^ N.D.	5.0-6.0	^c^ N.A.	N.A.	N.A.	N.A.	N.A.	This study
Q2	N.D.	6.5-7.5	F1	6.5-7.0	N1	6.5-7.0	cyclo(Tyr-Pro), C_14_H_16_N_2_O_3_	This study, [16]
^d^ Q^ns1^	N.D.	N.D.	F2	7.0-7.5	N2	7.0-7.5	cyclo(Ser-Pro), C_8_H_12_N_2_O_3_	This study, [16]
Q^ns2^	N.D.	N.D.	F3	7.5-7.8	N3	7.5-7.8	cyclo(Leu-Pro), C_11_H_18_N_2_O_2_	This study, [16]
Q^ns3^	N.D.	N.D.	F4	7.8-8.1	N4	7.8-8.1	cyclo(Leu-Pro), C_11_H_18_N_2_O_2_	This study, [16]
Q^ns4^	N.D.	N.D.	F5	8.1-9.0	N5	8.1-9.0	cyclo(Val-Pro), C_10_H_16_N_2_O_2_	This study, [36]
Q3	N.D.	9.0-10.0	F6	9.5-10.5	N6	9.5-10.5	cyclo(Tyr-Pro), C_14_H_16_N_2_O_3_	This study, [36]
Q4	N.D.	11.0-12.0	N.A.	N.A.	N.A.	N.A.	N.A.	This study
Q5	N.D.	12.5-13.0	F7	12.2-13.2	N7	12.2-13.2	*cis*-cyclo(L-Val-LPro), C_10_H_16_N_2_O_2_	This study, [36]
N.D.	N.D.	14.0-14.8	F8	14.0-15.0	N8	14.0-15.0	*cis*-cyclo(L-Leu-LHyp), C_11_H_18_N_2_O_3_	This study, [16]
Q6	N.D.	14.5-15.5	F9	15.0-16.5	N9	15.0-16.0	cyclo(Ser-Pro), C_8_H_12_N_2_O_3_	This study, [36]
Q7	N.D.	16.5-17.5	F10	17.0-18.5	N10	17.0-18.5	cyclo(Phe-Ala), C_12_H_14_N_2_O_2_	This study, [16]
Q8	N.D.	18.5-19.5	F11	19.0-20.0	N11	19.0-20.0	cyclo(Tyr-Pro), C_14_H_16_N_2_O_3_	This study, [16]
N.D.	N.D.	N.D.	F12	20.5-22.0	N12	20.5-22.0	cyclo(Leu-Pro), C_11_H_18_N_2_O_2_	This study, [36]
Q9	N.D.	22.0-23.0	F13	22.5-23.5	N13	22.5-23.5	*cis*-cyclo(L-Leu-LPro), C_11_H_18_N_2_O_2_	This study, [16]
Q10	N.D.	23.7-24.3	F14	24.0-24.5	N.A.	N.A.	cyclo(Met-Pro), C_10_H_16_N_2_O_2_S_1_	This study, [16]
Q11	N.D.	24.5-25.5	F15	24.5-25.0	N.A.	N.A.	DL-3-phenyllactic acid, C_9_H_10_O_3_	This study, [16]
Q12	N.D.	25.5-26.5	F16	26.0-27.0	N14	26.0-27.0	cyclo(Phe-Pro), C_14_H_16_N_2_O_2_	This study, [16]
Q13	N.D.	26.5-27.5	N.A.	N.A.	N.A.	N.A.	N.A.	This study
Q14	N.D.	30.5-31.5	F17	30.0-31.0	N15	30.0-31.0	*cis*-cyclo(L-Phe-LPro), C_14_H_16_N_2_O_2_	This study, [31]
Q15	N.D.	31.5-32.5	N.A.	N.A.	N.A.	N.A.	N.A.	This study

^a^Retention time (min).

^b^Non-detected or non-separable.

^c^Not applicable.

^d^Particularly non-separable.

**Table 4:** The results of mass spectrometric analysis of QE CDP fractions using EI/CI GC-MS.

Fraction	m/z of [M+1]^+^	m/z (%) of EI-MS	Reference or predicted molecules
Q6	185.0	55.2 (30.2), 57.2 (73.1), 69.2 (100.0), 70.3 (18.9), 71.3 (31.8), 83.3 (27.1), 98.3 (8.1), 99.3 (12.5), 113.4 (10.8), 127.4 (15.4), 129.4 (13.0), 141.5 (8.6), 155.5 (12.0), 183.6 (6.2)	*cis*-cyclo(Ser-Pro), C_8_H_12_N_2_O_3_ This study
Q9	211.1	55.0 (13.97), 68.0 (7.64), 69.0 (6.21), 70.1 (11.72), 71.1 (7.13), 83.0 (8.63), 86.0 (4.28), 91.0 (3.64), 98.0 (8.17), 111.0 (14.11), 126.0 (7.61), 153.0 (1.51), 154.0 (100.0), 155.0 (18.50), 167.0 (1.67), 195.0 (0.93)	*cis*-cyclo(L-Leu-L-Pro), C_11_H_18_N_2_O_2_ This study

**Table 5:** The antibacterial activity of QE fractions, specifically Q6 and Q9, and other unidentified fractions against both bacterial indicators and multidrug-resistant bacteria.

Type of pathogen/Experiment	Fraction							
	Q1	Q2	Q^ns^	Q3	Q4	Q5	Q6	Q7
^a,b,c,1^ MIC	^d^ N.D.	N.D.	199.5 ± 6.52	N.D.	N.D.	N.D.	N.D.	N.D.
^2^ MIC	N.D.	N.D.	N.D.	N.D.	N.D.	N.D.	N.D.	N.D.
^3^ MIC	N.D.	N.D.	230.0 ± 9.44	N.D.	N.D.	N.D.	N.D.	N.D.
^4^ MIC	N.D.	N.D.	260.0 ± 8.21	N.D.	N.D.	N.D.	N.D.	N.D.
^5^ MIC	N.D.	N.D.	N.D.	N.D.	N.D.	N.D.	N.D.	N.D.
^6^ MIC	N.D.	N.D.	221.0 ± 5.48	N.D.	N.D.	N.D.	N.D.	N.D.
-continued								
Type of pathogen/Experiment	Fraction							
	Q8	Q9	Q10	Q11	Q12	Q13	Q14	Q15
^a,b,c,1^ MIC	N.D.	11.6 ± 0.62	N.D.	N.D.	N.D.	N.D.	N.D.	N.D.
^2^ MIC	N.D.	13.0 ± 0.59	N.D.	N.D.	N.D.	N.D.	N.D.	N.D.
^3^ MIC	N.D.	10.0 ± 0.54	N.D.	N.D.	N.D.	N.D.	N.D.	N.D.
^4^ MIC	N.D.	23.0 ± 0.81	N.D.	N.D.	N.D.	N.D.	N.D.	N.D.
^5^ MIC	N.D.	21.0 ± 0.34	N.D.	N.D.	N.D.	N.D.	N.D.	N.D.
^6^ MIC	N.D.	14.0 ± 1.10	N.D.	N.D.	N.D.	N.D.	N.D.	N.D.

^a^Data are presented as mean ± standard error of the mean from three independent experiments.

^b^The KNIH provided indicator bacterial strains, including Gram-positive bacteria (^1^*B. subtilis* and ^2^*S. aureus*) and Gramnegative bacteria (^3^*E. coli*), as well as multidrug-resistant bacteria, including Gram-positive bacteria (^4^*S. aureus* 11471 and ^5^*S. pneumoniae* 14596) and Gram-negative bacteria (^6^*S*. Typhimurium 12219).

^c^MIC: Minimum inhibitory concentration (mg/l).

^d^Not detected.

**Table 6:** The antimicrobial activity of QCDPF2 (containing Q9) and QCDPF3 (containing Q6 and Q9) against pathogenic microorganisms.

	Fraction					
Type of pathogen/ Experiment	Q6 (*cis*-cyclo(L-Ser-L-Pro))	Q9 (*cis*-cyclo(L-Leu-L-Pro))	^c^ QCDPF1	^d^ QCDPF2	^e^ QCDPF3	^f^ QCDPF4
	^a,b^ MIC (mg/l)					
^m^ Multidrug-resistant bacteria						
*S. aureus* 11471^1^	^4^ N.D.	23.0 ± 0.81	N.D.	46.7 ± 0.94	36.32 ± 0.52	N.D.
*S. pneumoniae* 14596^2^	N.D.	21.0 ± 0.34	N.D.	41.6 ± 1.03	35.17 ± 0.63	N.D.
*S*. Typhimurium 12219^3^	N.D.	14.0 ± 1.1	N.D.	32.0 ± 1.12	24.32 ± 0.19	N.D.
	Fraction					
Type of pathogen/ Experiment	Q6 (*cis*-cyclo(L-Ser-L-Pro))	Q9 (*cis*-cyclo(L-Leu-L-Pro))	QCDPF1	QCDPF2	QCDPF3	QCDPF4
Active concentration of complex (mg complex in 3 mL agar assay)						
Fungi						
*G. boninense*	5.63 ± 0.91	4.98 ± 0.63	N.D.	14.26 ± 0.59	10.22 ± 0.53	N.D.
*C. albicans*	N.D.	N.D.	N.D.	13.64 ± 0.33	12.53 ± 0.21	N.D.
-continued						
	Fraction					
Type of pathogen/ Experiment	^g^ QCDPF5 (QCDPF1– Q6)	^h^ QCDPF6 (QCDPF2– Q9)	^i^ QCDPF7 (QCDPF3– Q6)	^j^ QCDPF8 (QCDPF3– Q9)	^k^ QCDPF9 (QCDPF1+ Q9)	^l^ QCDPF10 (QCDPF2+ Q6)
	^a,b^ MIC (mg/l)					
^m^ Multidrug-resistant bacteria						
*S. aureus* 11471^1^	^n^ N.D.	N.D.	38.16 ± 0.90	N.D.	45.71 ± 1.96	44.06 ± 2.34
*S. pneumoniae* 14596^2^	N.D.	N.D.	36.04 ± 0.87	N.D.	40.88 ± 0.84	39.22 ± 2.00
*S*. Typhimurium 12219^3^	N.D.	N.D.	24.72 ± 0.65	N.D.	33.20 ± 0.99	31.80 ± 1.54
	Fraction					
Type of pathogen/ Experiment	QCDPF5 (QCDPF1– Q6)	QCDPF6 (QCDPF2– Q9)	QCDPF7 (QCDPF3– Q6)	QCDPF8 (QCDPF3– Q9)	QCDPF9 (QCDPF1+ Q9)	QCDPF10 (QCDPF2+ Q6)
Active concentration of complex (mg complex in 3 mL agar assay)						
Fungi						
*G. boninense*	N.D.	N.D.	11.48 ± 0.36	N.D.	13.23 ± 0.47	15.61 ± 0.87
*C. albicans*	N.D.	N.D.	14.58 ± 0.74	N.D.	14.35 ± 0.85	15.22 ± 0.64

^a^MIC: Minimum inhibitory concentration

^b^Data are presented as mean ± standard error of the mean from three independent experiments.

^c^QCDPF1; Q1+Q2+Q^ns1-ns4^+Q3+Q4+Q5+Q6 ((*cis*-cyclo(L-Ser-L-Pro))

^d^QCDPF2; Q7+Q8+Q10+Q11+Q12+Q13+Q14+Q15+Q9 (*cis*-cyclo(L-Leu-L-Pro))

^e^QCDPF3; QCDPF1+QCDPF2 (QCDPF3 contains Q6 ((*cis*-cyclo(L-Ser-L-Pro))) and Q9 (*cis*-cyclo(L-Leu-L-Pro))

^f^QCDPF4; QCDPF1+QCDPF2–Q6(*cis*-cyclo(L-Ser-L-Pro))–Q9(*cis*-cyclo(L-Leu-L-Pro))

^g^QCDPF5; QCDPF1–Q6 (*cis*-cyclo(L-Ser-L-Pro))

^h^QCDPF6; QCDPF2–Q9 (*cis*-cyclo(L-Leu-L-Pro))

^i^QCDPF7; QCDPF3–Q6 (*cis*-cyclo(L-Ser-L-Pro))

^j^QCDPF8; QCDPF3–Q9 (*cis*-cyclo(L-Leu-L-Pro))

^k^QCDPF9; QCDPF1+Q9 (*cis*-cyclo(L-Leu-L-Pro))

^l^QCDPF10; QCDPF2+Q6 (*cis*-cyclo(L-Ser-L-Pro))

^m^The KNIH provided multidrug-resistant Gram-positive bacteria (^1^ *S. aureus* 11471 and ^2^ *S. pneumoniae* 14596) and Gramnegative bacteria (^3^ *S*. Typhimurium 12219).

^4^Not detected.
