# Supplementary material for: Antimicrobial Cyclic Dipeptides from Japanese Quail (Coturnix japonica) Eggs Supplemented with Probiotic Lactobacillus plantarum
Source: J Microbiol Biotechnol. 2023 Dec 19;34(2):314–29. doi: 10.4014/jmb.2311.11006 (PMC10940788; doi:10.4014/jmb.2311.11006)
Supplement: Supplementary file 1 [file jmb-34-2-314-supple.pdf]

J. Microbiol. Biotechnol.
https://doi.org/10.4014/jmb.2311.11006

Supplementary Tables

Antimicrobial cyclic dipeptides from Japanese quail (*Coturnix japonica*) eggs supplemented with probiotic *Lactobacillus plantarum*

Sa-Ouk Kang[1,3], Min-Kyu Kwak[2,*]

[1] *Laboratory of Biophysics, School of Biological Sciences, and Institute of Microbiology, Seoul National University, Seoul 08826, Republic of Korea*

[2] *Laboratory of Microbial Physiology and Biotechnology, Department of Food and Nutrition, College of Bio-Convergence, and Institute of Food and Nutrition Science, Eulji University, Seongnam 13135, Republic of Korea*

[3] Present address: Irwee Institute, B-503, Bundang Techno Park, 744, Pangyo-ro, Bundang-gu, Seongnam-si, Gyeonggi-do 13510, Republic of Korea

* Corresponding author at:

E-mail: genie6@eulji.ac.kr (M.-K. Kwak).

Supplementary Tables

Table S1. A comparison between the retention behaviors (t_R) of K10N and/or K10S, in relation to those of LBP-K10 and LBP-K06, using HPLC chromatographic separation.

Fraction	m/z of $[M+1]^+$	m/z (%) of EI-MS	Reference or predicted molecules
Q1	[a] N.D.	N.D.	Disappeared fraction
Q2	N.D.	N.D.	Non-separable fraction
Q^{ns1}	N.D.	N.D.	Overlapped and non-separable fraction
Q^{ns2}	N.D.	N.D.	Overlapped and non-separable fraction
Q^{ns3}	N.D.	N.D.	Overlapped and non-separable fraction
Q^{ns4}	N.D.	N.D.	Overlapped and non-separable fraction
Q3	N.D.	N.D.	Overlapped, elution time shifted, and/or low s/n ratio
Q4	N.D.	N.D.	Overlapped, elution time shifted, and/or low s/n ratio
Q5	N.D.	N.D.	Overlapped and non-separable fraction
Q6	185.0	cyclo(Ser-Pro), $C_8H_{12}N_2O_3$	This study, [1]

Q7	N.D.	N.D.	Disappeared fraction
Q8	N.D.	N.D.	Non-separable fraction
Q9	211.0	*cis*-cyclo(L-Leu-L-Pro), $C_{11}H_{18}N_2O_2$	This study, [2]
Q10	N.D.	N.D.	Non-separable fraction
Q11	N.D.	N.D.	Overlapped, elution time shifted, and/or low s/n ratio
Q12	N.D.	N.D.	Overlapped, elution time shifted, and/or low s/n ratio
Q13	N.D.	N.D.	Overlapped, elution time shifted, and/or low s/n ratio
Q14	N.D.	N.D.	Overlapped and non-separable fraction
Q15	N.D.	N.D.	Disappeared fraction

[a] Non-detected or non-separable.

Table S2. The antimicrobial activity of individual CDPs (Q6 and Q9), as well as other fractions, against pathogenic fungi using K10S as the model.

Type of pathogen	Fraction							
	[a] Active concentration of complex (mg complex in 3 mL agar assay)							
	Fraction							
	Q1	Q2	Q^{ns}	Q3	Q4	Q5	Q6	Q7
	[a] Active concentration of complex (mg complex in 3 mL agar assay)							
G. boninense	[b] N.D.	N.D.	N.D.	N.D.	N.D.	N.D.	5.63 ± 0.91	N.D.
C. albicans	N.D.	N.D.	N.D.	N.D.	N.D.	N.D.	N.D.	N.D.
	Fraction							
	Q8	Q9	Q10	Q11	Q12	Q13	Q14	Q15
	[a] Active concentration of complex (mg complex in 3 mL agar assay)							
G. boninense	N.D.	4.98 ± 0.63	N.D.	N.D.	N.D.	N.D.	N.D.	N.D.
C. albicans	N.D.	N.D.	N.D.	N.D.	N.D.	N.D.	N.D.	N.D.

[a] Data are presented as mean ± standard error of the mean from three independent experiments.

[b] Not detected.

References

1. Kwak M-K, Liu R, Kim M-K, Moon D, Song S-H, Kang S-O. 2014. Cyclic dipeptides from lactic acid bacteria inhibit the proliferation of pathogenic fungi. *J. Microbiol.* **52:** 64-70.
2. Kwak M-K, Liu R, Kwon J-O, Kim M-K, Kim AH, Kang S-O. 2013. Cyclic dipeptides from lactic acid bacteria inhibit the proliferation of influenza A virus. *J. Microbiol.* **51:** 836-843.
